# Idiopathic Intracranial Hypertension With Sustained Normal Opening Pressure: A Case Report

**DOI:** 10.7759/cureus.87940

**Published:** 2025-07-14

**Authors:** Stanley Pierre, Shantell Steele, Melissa Candela, Allison Rojas, Nayle Araguez-Ancares

**Affiliations:** 1 Surgery, Ross University School of Medicine, St. Michael, BRB; 2 Surgery, Ross University School of Medicine, Miramar, USA; 3 Surgery, American University of the Caribbean School of Medicine, Cupecoy, SXM; 4 Internal Medicine, Jackson Memorial Hospital, Miami, USA

**Keywords:** bilateral optic neuritis, intracranial idiopathic hypertension, myelinating oligodendrocyte glycoprotein antibody-associated disease, myelin-oligodendrocyte glycoprotein (mog), myelin sheath

## Abstract

Idiopathic intracranial hypertension (IIH) is a clinical diagnosis made from the exclusion of other possible causes of increased intracranial pressure (ICP): cerebrospinal fluid (CSF) overflow obstruction, mass lesion, infection, or malignancies. IIH is a phenomenon with a rapid onset that most notably presents in obese women of childbearing age. We present the case of a 30-year-old African American woman with a past medical history of bipolar disorder who arrived at the emergency department (ED) with acute bilateral vision loss, two weeks of bilateral retro-orbital headache, painful eye movements, and progressive vision loss with a decrease in appetite. Management involved plasma exchange, high-dose steroids, consistent neurological and ophthalmic tests, and close observation and follow-up. One week after the most recent ED visit, the patient confirmed a significant improvement in vision. When diagnosing our patient with IIH, it was imperative to rule out myelinating oligodendrocyte glycoprotein antibody-associated disease (MOGAD), multiple sclerosis (MS), and other demyelinating diseases such as optic neuritis. Initial brain imaging using computed tomography (CT) without contrast showed no evidence of intracranial hemorrhage but demonstrated the presence of prominent sheath complexes, suggestive of optic neuritis, and a slightly small pituitary gland height for a patient who is 30 years of age. Further testing with a CT venogram (CTV) and a magnetic resonance imaging (MRI) of the head and neck confirmed demyelination. The patient's lumbar puncture continuously revealed a normal opening pressure during her hospital stay. This case presentation will highlight and compare the current literature regarding the clinical presentation and management of idiopathic intracranial hypertension.

## Introduction

Idiopathic intracranial hypertension (IIH) is characterized by increased intracranial pressure (ICP) without anatomical deformities or obstructions. It is commonly seen in obese women who are in their childbearing years. Diagnostic workups may be normal, other than increased cerebrospinal fluid (CSF) pressure [1]. Interestingly, our case of a 30-year-old female patient exhibits these characteristics, with an atypical finding of normal cerebrospinal fluid pressure. Common findings in neuroimaging of IIH may include smooth-walled venous stenosis, fully unfolded optic nerve sheaths, and flattened globes [1]. Overall, the annual incidence is higher in the Middle East, in contrast to other regions of the world, such as Japan [1,2]. There is also a parallel rise in IIH with obesity in women [3].

Regarding the risks associated with IIH, given the parallel connection between the prevalence of the disease and the rise in obesity, it is said to be a major risk factor. In addition, gender is another major risk factor, as 90% of cases are women, particularly in the postpuberty age group [1]. It is also noted that although prevalence may not be different between African Americans and Caucasians, African American patients with IIH may have more severe visual outcomes [1]. IIH has also been seen with some endocrinological disorders such as Addison's disease, hypoparathyroidism, and steroid withdrawal [1]. In this report, we aim to discuss the current literature in combination with the presentations and clinical findings seen regarding the rare presentation of IIH.

## Case presentation

We present the case of a 30-year-old, obese African American woman with a past medical history of bipolar disorder who arrived at the emergency department (ED) with a two-week duration of headaches, painful eye movements, and progressive vision loss. The headache was described as a migraine, pressure-like pain with a left-sided predominance and rated 8/10 in severity, accompanied by pain associated with lateral eye movements. Two weeks prior, the patient visited the ED with blurry vision and a headache, and was discharged with 800 mg of ibuprofen with follow-up to a local eye institute. A brain computed tomography (CT) was performed at this time, revealing no acute intracranial abnormalities, along with a partially empty sella and small ventricles, findings suggestive of IIH. After the eye institute confirmed severe papilledema, the patient was prescribed Diamox 500 mg twice daily (BID) with the presumed diagnosis of IIH. Magnetic resonance imaging (MRI) could not be obtained because of body piercings, but papilledema continued to worsen. Given her physical examination findings of severe grade 4-5 papilledema and no light perception in either eye, she was sent to the ED for an urgent lumbar puncture, which demonstrated an opening pressure of 23. She confirms no previous history of neurological deficits prior to these series of events. Family history is notable for type 1 diabetes mellitus in her mother, a sister with thyroid disease, and a cousin with lupus.

On initial physical examination, blood pressure was 123/73 mmHg, with a heart rate of 67 bpm, respiratory rate of 18 breaths per minute, and an oxygen saturation of 97%. The patient was fully alert and in no acute distress. She confirmed a rapid decrease in vision from 70% to about 25%-40%. The patient stated that she was able to see objects at close range while also noticing a difference in color hues, with an inability to identify the color itself. Neurology was consulted and started the patient on high-dose IV methylprednisolone as soon as MRI results were available and revealed findings that suggest acute optic neuritis. The patient noted improvement in her baseline after three days of treatment. A lumbar puncture was also performed, which showed a normal opening pressure with a negative oligoclonal IgG bands test, negative anti-myelin-associated glycoprotein antibody (MAG) test, and negative neuromyelitis optica/Aquaporin-4 (NMO) antibody test. These are all highly specific antibodies for multiple sclerosis (MS), myelinating oligodendrocyte glycoprotein antibody-associated disease (MOGAD) [4], and neuromyelitis optica (NMOSD), respectively [5]. Alongside the treatment with steroids, the lumbar puncture resolved the patient's bilateral retro-orbital headache. There were no other neurological deficits noted, aside from the vision impairment.

Imaging was conducted to gain insight into possible differentials and evaluate the extent of the patient's condition. Initial imaging via brain CT without contrast showed no evidence of intracranial hemorrhage but demonstrated the presence of prominent sheath complexes, also suggestive of optic neuritis, although CT is limited in its diagnosis. It was also noted that the height of the pituitary gland was slightly smaller for the patient's age. CT venogram (CTV) was performed thereafter, which further showed tortuous and enlarged bilateral optic nerves with flattening of the optic discs bilaterally. In addition, the CTV showed moderate to severe diffuse narrowing of the transverse and sigmoid sinuses bilaterally, with focal narrowing suspected at the transverse sinus/sigmoid junctions. These constellations of findings are suggestive of intracranial hypertension. An MRI of the orbits/face/neck with and without contrast found evidence of acute optic neuritis along with demyelinating lesions suggestive of MOGAD. MRI of the cervical (C) and thoracic (T) spine without contrast showed no evidence of demyelinating disease, while the magnetic resonance venography (MRV) showed focal narrowing of the lateral transverse sinuses bilaterally with suggestion of papilledema, raising the possibility of superimposed intracranial hypertension. After the diagnostic tests and blood work were complete, plasma exchange therapy was initiated. The plasma exchange sessions included administration of IV corticosteroids every other day starting from day 2 of admission for a total of five sessions of high-dose steroids over 12 days. Her vision loss initially improved, plateaued, and improved again throughout this treatment course. The patient was discharged with 60 mg of methylprednisolone and 40 mg of Protonix daily, along with patient education and guidance on weight loss to decrease body mass index (BMI). Her diagnosis at the time of discharge was IIH along with a working diagnosis of associated antibody-negative NMO. She is to follow up with a local neurology clinic for further management.

**Figure 1:**
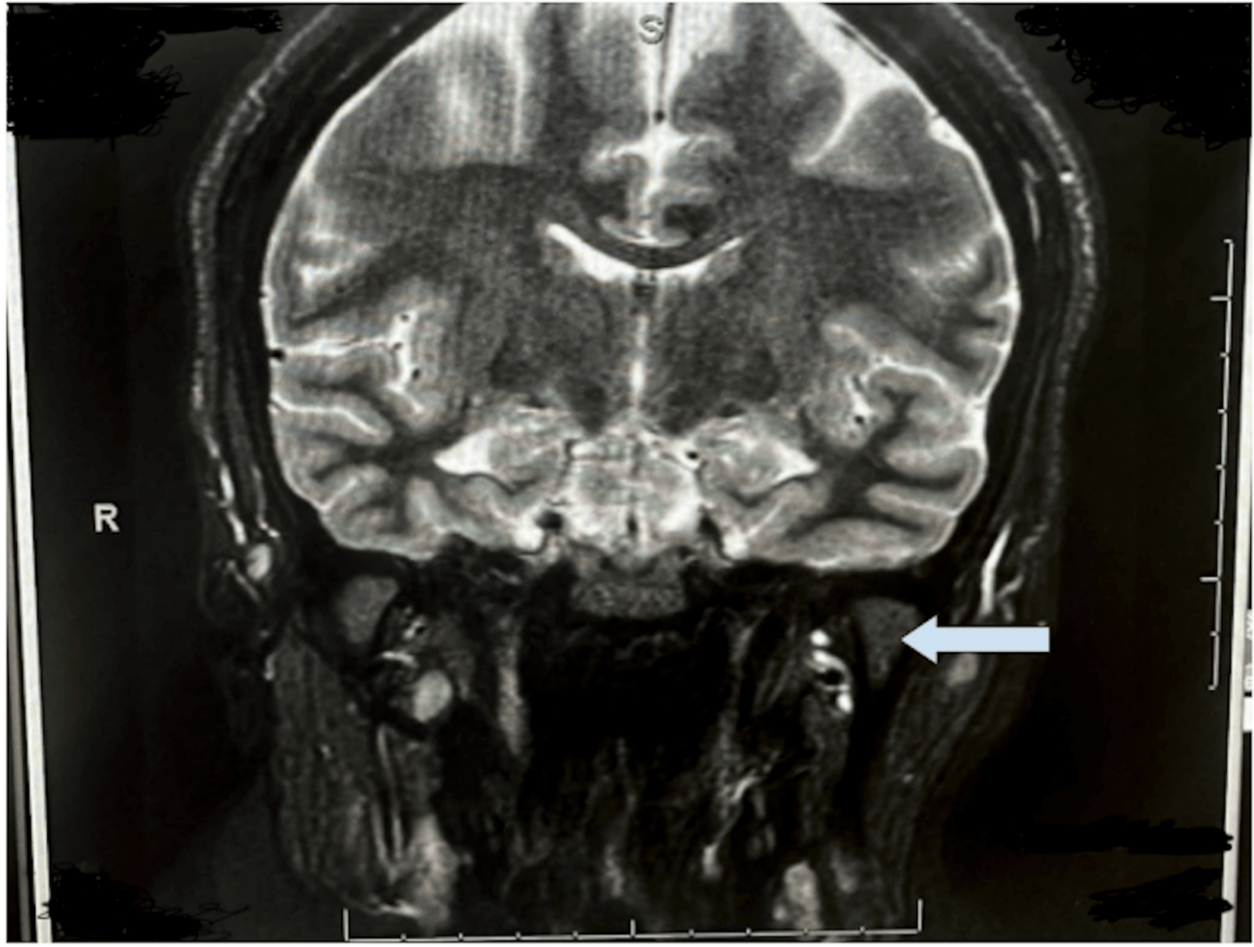
MRI of the orbits Blue arrow highlighting optic neuritis MRI: magnetic resonance imaging

**Figure 2:**
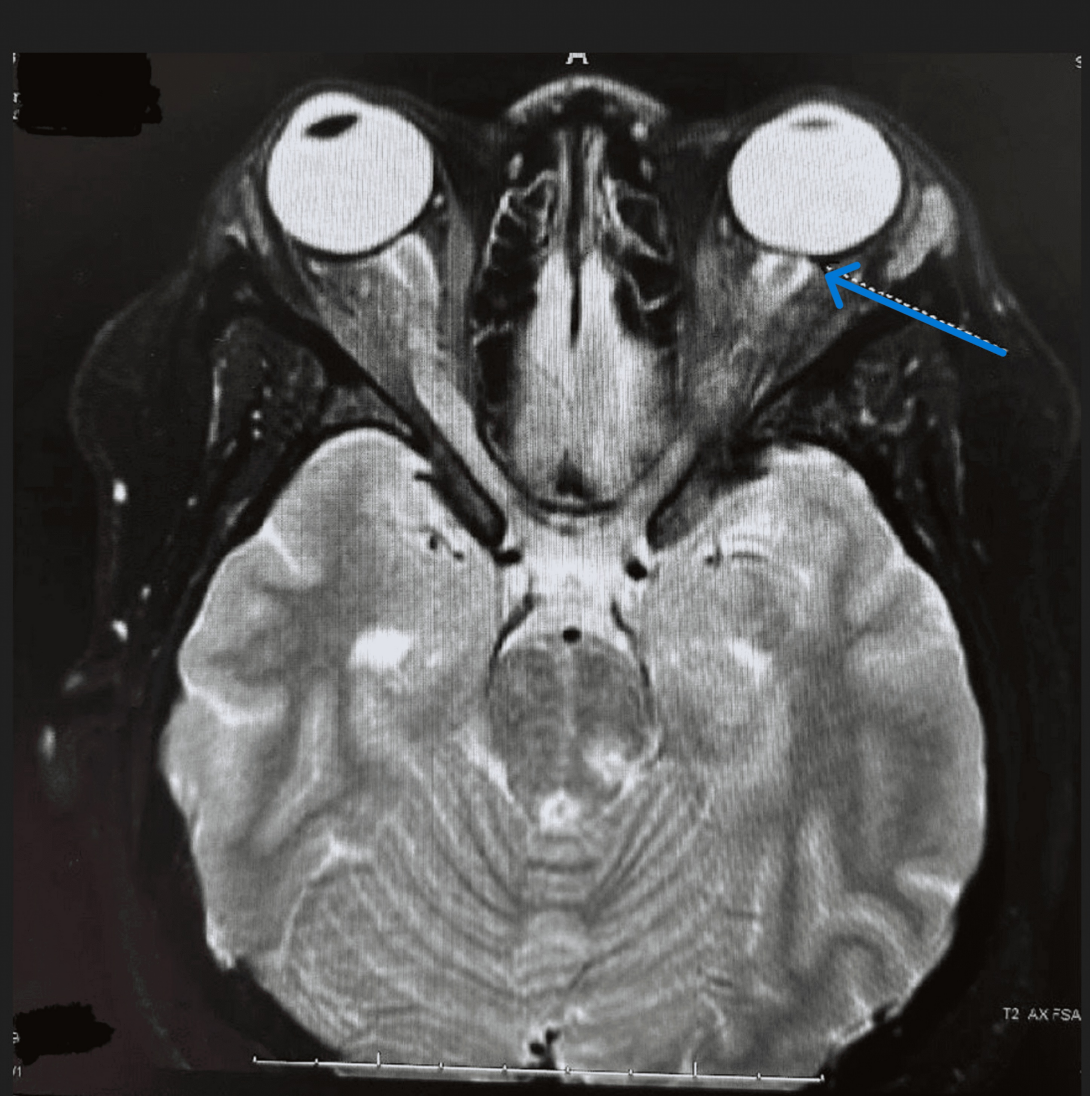
MRI of the brain: tortuous right optic nerve Blue arrow highlighting increased signal of the left optic nerve MRI: magnetic resonance imaging

Table 1 and Table 2 present the results of the slit lamp examination and fundus examination, respectively. Figure 1 and Figure 2 show the MRI images of the orbits and brain, respectively.

**Table 1:** Slit lamp examination

Anatomy location	Right	Left
Lids/lashes	Normal	Normal
Conjunctiva/sclera	White and quiet	White and quiet
Cornea	Clear	Clear
Anterior chamber	Deep and quiet	Deep and quiet
Iris	Round and flat	Round and flat
Lens	Clear	Clear

**Table 2:** Fundus examination

Fundus	Right	Left
Vitreous	Normal	Normal
Disc	360 severe edema with vessel obscuration and heme	360 severe edema with vessel obscuration and heme
Macula	Normal	Normal
Vessels	Normal	Normal

## Discussion

Previous literature on idiopathic intracranial hypertension (IIH) in a young African American female patient shows that although an infrequent phenomenon, it is common in obese women of childbearing age [6]. IIH often manifests with two prominent symptoms, the first being progressive vision loss resulting from papilledema and chronic headache [2,6]. Additional manifestations can include cranial nerve palsies, cognitive deficits, tinnitus, and olfactory dysfunction [6]. Extensive imaging and laboratory studies are done to rule out other demyelinating causes of vision impairment, chronic headache, and changes in appetite. A normal opening pressure on lumbar puncture in idiopathic intracranial hypertension is uncommon, but the risk factors of this patient and findings on the CT venogram further prove idiopathic intracranial hypertension. Although antibodies were negative for MOGAD, MS, and NMOSD, a patient can have seronegative neuromyelitis optica, which should be further investigated. Treatment of idiopathic intracranial hypertension varies by case presentation and focuses on treating the underlying disease, minimizing the headache burden, and protecting vision [6,7]. The mainstay treatment focuses on weight loss accompanied by the administration of acetazolamide, which lowers ICP in most cases [6]. Surgery is explored when there is a rapid or progressive decline in visual function [6]. Surgical treatment includes CSF shunting, venous sinus stenting, and optic nerve sheath fenestration [6]. In this specific case, there was evidence of a concurrent disease process that led to an acute decompensation of the patient's existing IIH. As a result, our patient was treated with steroids initially and maintained on steroids during her hospital stay and upon discharge. Plasma exchange sessions were initiated, with administration of IV corticosteroids every other day. In total, the patient was treated with five sessions of plasma exchange and high-dose steroids for 12 days, with a gradual improvement of vision.

## Conclusions

Idiopathic intracranial hypertension is a diagnosis made from exclusion, with risk factors including obesity and women of childbearing age. In this case report, we presented a 30-year-old African American woman with progressive bilateral vision loss with a bilateral retro-orbital, migraine-like headache. CTV showed findings suggestive of optic neuritis, papilledema, and superimposed intracranial hypertension. Lastly, on CT of the brain and MRI examination of the orbit/face/neck with and without contrast, findings suggested acute optic neuritis. With these radiological findings, it is concluded that imaging should not be delayed as it is an important step in diagnosing IIH or ruling out demyelinating diseases and other medical emergencies. Doing so may delay appropriate treatment and lead to worse outcomes, especially if other disease processes are at play.

In addition, the patient's CSF maintained an unusual finding of consistent normal opening pressure, which supports why imaging studies are also vital to provide further intervention. Although the results of the lumbar puncture showed a normal opening pressure, this should not prolong a diagnosis of idiopathic intracranial hypertension because CSF pressures tend to fluctuate throughout the day. Supporting evidence found through fundoscopic examination and radiology results, such as papilledema and involvement of the optic nerve, with symptoms of headache and vision loss, led the medical team to assemble a diagnosis of idiopathic intracranial hypertension. Although testing for other autoimmune diseases was negative, it must be noted that there is a recognized association specifically between NMOSD and IIH. IIH can occur alongside or be a presentation of NMOSD, which may be the case in this particular instance.
